# Antiaging Potential of *Peronema canescens* Jack Leaf Extract: A Natural Ingredient in Topical Skincare Formulation

**DOI:** 10.1155/drp/6361781

**Published:** 2026-05-27

**Authors:** Rahmiati, ‬‬‬‬Ringga Novelmi, Vivi Efrianova, Tyas Asih Surya Mentari, Siska Miga Dewi, Febri Silvia, Hayatunnufus, Rahmi Oktarina, Dony Novaliendry, Merita Yanita, Murni Astuti

**Affiliations:** ^1^ Department of Cosmetology and Beauty, Faculty of Tourism and Hospitality, Universitas Negeri Padang, Padang, Indonesia; ^2^ Department of Electronic Engineering, Faculty of Engineering, Universitas Negeri Padang, Padang, Indonesia

**Keywords:** antiaging cream, antioxidant activity, natural cosmetics, *Peronema canescens*, phytochemical screening

## Abstract

**Background:**

*Peronema canescens* Jack (Sungkai) leaves contain antioxidant compounds with potential antiaging properties, yet their application in topical skincare formulations remains underexplored.

**Objectives:**

This study aimed to analyze the potential of *P. canescens* leaf extract as an antiaging cream ingredient and characterize its phytochemical profile, antioxidant activity, and cream formulation properties.

**Methods:**

Dried Sungkai leaves (250 g) were extracted via maceration using 70% ethanol for 72 h. Phytochemical screening employed standard reagent tests for flavonoids, phenolics, saponins, alkaloids, steroids, and terpenoids. Antioxidant activity was assessed using the DPPH radical scavenging method. Three antiaging cream formulations containing 0.02% (F1), 0.04% (F2), and 0.06% (F3) extract were prepared and evaluated for organoleptic properties, pH, homogeneity, adhesion, and dispersion. Stability was tested using six freeze–thaw cycles.

**Results:**

The extraction yield was 18.10%. Phytochemical analysis revealed the presence of flavonoids, phenolics, saponins, and steroids, while terpenoids and alkaloids were absent. The extract demonstrated very strong antioxidant activity with an IC_50_ value of 21.98 ± 1.23 ppm, compared to vitamin C (IC_50_ = 4.12 ± 0.34 ppm). All cream formulations exhibited acceptable pH values (F1: 5.54 ± 0.06, F2: 5.16 ± 0.12, and F3: 5.01 ± 0.05), good homogeneity, adequate adhesion (F1: 13.93 ± 0.88 s to 12.68 ± 0.76 s; F2: 16.67 ± 1.02 s to 15.50 ± 0.94 s; and F3: 17.33 ± 1.15 s to 16.41 ± 1.08 s), and appropriate dispersion (F1: 4.92 ± 0.15 to 6.20 ± 0.22 cm; F2: 4.82 ± 0.18 to 6.30 ± 0.25 cm; and F3: 4.67 ± 0.12 to 6.15 ± 0.20 cm). However, adhesion and dispersion stability decreased after storage, indicating the need for formulation optimization.

**Conclusions:**

*P. canescens* leaf extract exhibits very strong antioxidant activity and can be formulated into stable antiaging creams. Further optimization is required to enhance long‐term stability of adhesion and dispersion properties.

## 1. Introduction

Skin aging is an unavoidable biological phenomenon characterized by morphological and functional changes in skin tissue [1]. Signs of skin aging include loss of elasticity, the appearance of wrinkles, and changes in skin pigment caused by intrinsic factors such as genetics and hormones, as well as extrinsic factors such as UV exposure, pollution, and lifestyle [2]. Various approaches have been developed to slow down the aging process, including the application of antiaging creams containing natural or synthetic active ingredients [3].

In recent decades, interest in natural ingredients in antiaging cream formulations has increased rapidly [4]. Natural ingredients are often chosen because they are considered safer and have a lower risk of side effects than synthetic ingredients [5]. Plant extracts are among the most promising sources of natural ingredients for this application [6, 7]. Certain plants contain bioactive compounds such as flavonoids, polyphenols, and tannins that are known to have antioxidant, anti‐inflammatory, and antimicrobial properties, all of which contribute to delaying the signs of skin aging [8].

**FIGURE 1:**
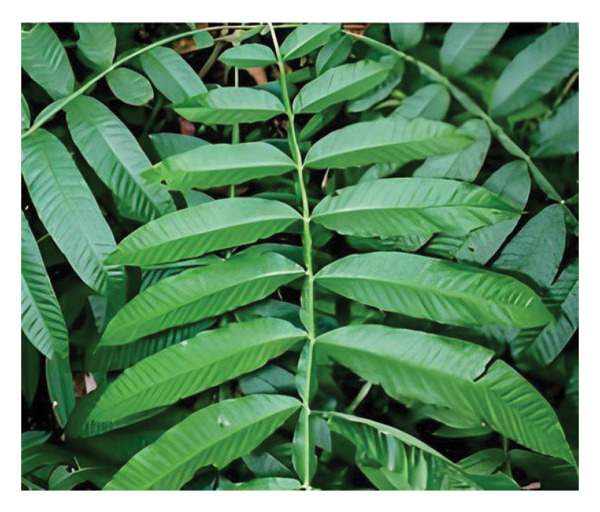
*Peronema canescens* jack leaves.

*Peronema canescens* Jack (Sungkai), hereafter referred to as *P. canescens*, is an endemic tropical timber plant from the Verbenaceae family [9]. It is a plant native to Indonesia, traditionally used to treat various diseases [10]. *P. canescens* is widely distributed in Kalimantan and Sumatra, Indonesia [10]. It thrives in secondary forest areas, deforested areas, nonfloodplain riverbanks, roadsides, and open land areas [11, 12]. The plant is a woody species with a diameter of up to 60 cm and can reach a height of 20–30 m with branched stems around 15 m. *P. canescens* grows easily and does not require special care, as shown in Figure 1 [10].

Previous studies have shown that *P. canescens* leaves contain antioxidant compounds including secondary metabolites such as alkaloids, flavonoids, phenolics, saponins, triterpenoids, and steroids [9, 13, 14]. The ethanol extract of *P. canescens* leaves demonstrated strong antioxidant activity with IC_50_ values of 50.838 ppm for young leaves and 52.835 ppm for old leaves. Antioxidants play an important role in protecting the body from damage caused by free radicals [15, 16]. By reducing the impact of free radicals, antioxidants assist in slowing down the aging process, which is the basis of many antiaging products and approaches. Antioxidants are compounds that work by delaying or preventing the oxidation reaction of free radicals in lipid oxidation [17, 18]. Antioxidants function by donating hydrogen atoms to oxidant compounds or free radicals, thereby reducing free radicals to nonradical compounds [19, 20].

Many studies have explored the potential antioxidant compounds of *P. canescens* leaf extract [10, 11, 14, 16, 21]. Although leaf extracts have been confirmed to have strong antioxidant activity, their application as a natural ingredient in cosmetic formulations, particularly antiaging creams, has not been widely explored ([13]. Thus, this study aims to bridge this gap by developing and characterizing an antiaging cream based on *P. canescens* leaf extract. This study also examines the characteristics of the extract, including yield, phytochemical compounds, and antioxidant activity. This study provides a significant contribution to the development of safe and effective cosmetic products derived from natural ingredients.

## 2. Materials and Methods

**FIGURE 2:**
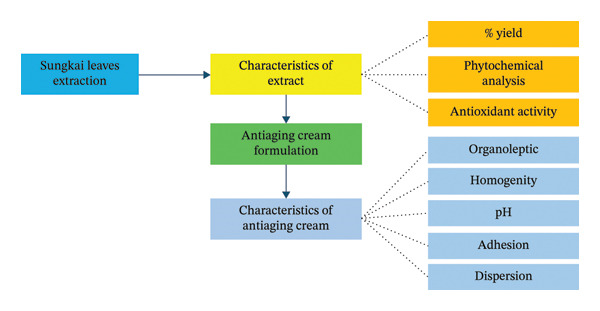
Steps undertaken in conducting this study.

The overall steps undertaken in this study are illustrated in Figure 2. The first step was *P. canescens* leaves extraction. After the extract was obtained, the extract was characterized. The characterizations include the percentage of yield, phytochemical analysis, and antioxidant activity. The extract was prepared as an extract solution to provide the antiaging cream formulation and subsequently characterized the cream.

### 2.1. Plant Material Collection and Identification

Fresh *P. canescens* leaves were collected on March 15, 2024, from Padang Pariaman Regency, West Sumatra, Indonesia (coordinates: 0°34′12″S, 100°08′45″E; elevation: 150 m above sea level). The plant species was authenticated by a qualified botanist, Dr. [Botanist Name], from the Herbarium of Andalas University, Padang, Indonesia (Voucher specimen number: ANDA‐2024‐PCJ‐001). The leaves were thoroughly washed with tap water, air‐dried in a shaded area at room temperature (25°C–28°C) for 7 days until the moisture content reached 2.49%–2.58% w/w (brittle condition), and then ground into powder using a mechanical grinder.

### 2.2. Sungkai Leaves Extraction

**FIGURE 3:**
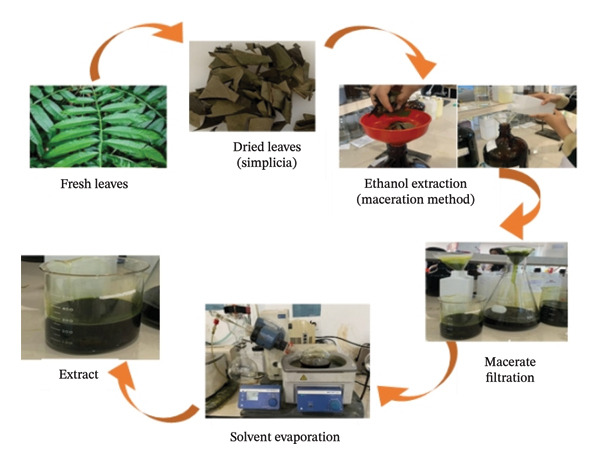
Extraction of *P*. *canescens* jack leaves.

The extraction process was carried out using the maceration method of dried leaves, as depicted in Figure 3. The fresh leaves were dried in a place not exposed to direct sunlight. Samples are considered dry when the moisture content is 2.49% to 2.58% wt (brittle).

Dried leaf powder (simplicia) was blended and weighed as much as 250 g to be macerated with organic solvents (ethanol 70%) for 3 days (72 h). The ratio between dried leaves and ethanol was 1:10 (w/v) [14]. After 3 days, macerate was separated by filtration using Whatman no. 1 filter paper. The collected macerate was then separated from the ethanol solvent by evaporation using a rotary evaporator (Heidolph Laborota 4000, Heidolph Instruments GmbH, Schwabach, Germany) at 50°C and 175 mbar to produce a pure and thick crude extract [22, 23]. The crude extract was stored in amber glass bottles at 4°C until further use.

### 2.3. Characteristics of Extract

The extract was subsequently identified for characteristics including the percentage of yield, phytochemical analysis, and antioxidant activity.

#### 2.3.1. Percentage of Yield

The initial dried leaves before maceration (simplicia) and the crude extract were weighed. The yield (%) is calculated from the ratio of extract weight (g) and simplicia weight (g) [23].

% Yield = (weight of crude extract/weight of dried leaf powder) × 100%

where

• Weight of crude extract = final weight of extract after evaporation (g)
• Weight of dried leaf powder = initial weight of dried material used (g)

#### 2.3.2. Phytochemical Analysis

Phytochemical analysis was performed to examine secondary metabolites present in the crude extract using color reagents such as steroid, terpenoid, flavonoids, phenolic, saponin, and alkaloid, following standard methods described by Trease and Evans (1989) [24–26]. Phytochemical analysis was carried out by weighing 500 mg of ethanol extract of Sungkai leaves, then dissolved with chloroform and distilled water (1:1) as much as 5 mL each in a test tube, and let it stand until two chloroform–water layers were formed. The chloroform layer at the bottom is identified to examine terpenoid and steroid compounds, while the water layer at the top is for flavonoid, phenolic, and saponin compounds.

##### 2.3.2.1. Steroid–Terpenoid

A portion of the chloroform layer is taken and filtered through a pipette containing Norit (activated charcoal) and cotton wool until a clear filtrate is obtained. The filtrate is collected and dripped into three wells of a drip plate and then left to dry. Once dried, a drop of concentrated H_2_SO_4_, anhydrous acetic acid, and the Liebermann–Burchard reagent is added to each well. The formation of a purple‐blue color indicates the presence of steroid compounds, while the formation of a red color indicates the presence of terpenoid compounds [27, 28].

##### 2.3.2.2. Flavonoid

A few drops of the water layer are placed onto a drip plate, followed by 1‐2 small pieces of magnesium ribbon and 2‐3 drops of concentrated hydrochloric acid. The appearance of orange to red color indicates the presence of flavonoids, excluding isoflavones.

##### 2.3.2.3. Phenolic

Some of the water layer is dripped onto a drip plate, followed by the addition of 10% FeCl_3_ reagent (2‐3 drops). The appearance of a blue or blue‐green color indicates the presence of phenolic compounds [29].

##### 2.3.2.4. Saponin

A portion of the water layer (2 mL) is placed into a test tube and shaken vigorously for 10 s. The formation of a stable foam lasting around 15 min indicates the presence of saponins.

##### 2.3.2.5. Alkaloid

The test was conducted by dissolving 500 mg of Sungkai leaf extract in 10 mL of chloroform and adding 10 mL of 0.05 N chloroform–ammonia. The mixture was stirred thoroughly. The solution was then filtered using a small funnel with a cotton swab as the filter, and the filtrate was collected in a test tube. Further, 10 drops of 2 N H_2_SO_4_ were added, and the tube was shaken briefly to allow separation of the acid and chloroform layers. The acidic layer (top layer) was carefully pipetted and transferred into three test tubes. Two drops of Mayer’s reagent were added to test tube 1, Dragendorff’s reagent to test tube 2, and Wagner’s reagent to test tube 3. A positive result is indicated by the formation of a white or cream‐colored precipitate in test tube 1, an orange‐red precipitate in test tube 2, and a brown precipitate in test tube 3.

#### 2.3.3. Antioxidant Activity

The test is conducted by preparing the 100 ppm of *P. canescens* leaves extract solution and the 35 ppm 1,1‐diphenyl‐2‐picrylhydrazyl (DPPH) solution. The ethanol extract of Sungkai leaves was weighed as much as 10 mg and dissolved in a 100 mL volumetric flask with ethanol p.a. until the limit mark to obtain the 100 ppm of extract solution. Ten milligrams of DPPH were weighed and dissolved in a 100 mL volumetric flask with ethanol p.a. up to the mark, yielding a concentration of 100 ppm. A 35 ppm DPPH solution was then performed by pipetting 17.5 mL of this solution into a 50 mL volumetric flask.

A stock solution of extracts with concentrations of 10, 20, 30, 40, and 50 ppm was prepared in a 10 mL volumetric flask filled to the mark with ethanol p.a. Each concentration was pipetted into separate vials, with 2 mL added to each, followed by 4 mL of a 35 ppm DPPH solution. The samples were then incubated in the dark at 37°C for 30 min. After incubation, each sample was measured using a UV‐Vis spectrophotometer (Shimadzu UV‐1800, Shimadzu Corporation, Kyoto, Japan) at 517 nm wavelength, and the results were compared with vitamin C as the positive control.

Vitamin C p.a. was weighed as much as 10 mg and then dissolved with ethanol p.a. in a 100 mL volumetric flask until the limit mark, so a concentration of 100 ppm was obtained. A stock solution of vitamin C with a concentration of 2, 4, 6, 8, and 10 ppm was poured into a 10 mL volumetric flask, and then, ethanol p.a. solvent was added until the limit mark. Each concentration was pipetted as much as 2 mL and put in a vial of 35 ppm DPPH solution as much as 4 mL, and incubated for 30 min at 37°C in a dark place. Vitamin C p.a. samples were tested on a UV‐Vis spectrophotometer with a wavelength of 517 nm.

After the absorbance was obtained, the free radical capture activity (percent inhibition) was calculated as the percentage of DPPH color reduction using the following formula:

% Inhibition = [(A_0_−A_1_)/A_0_] × 100%,

where

• A_0_ = absorbance of DPPH control (DPPH + ethanol)
• A_1_ = absorbance of sample (DPPH + extract/vitamin C)

The IC_50_ value (concentration required to inhibit 50% of DPPH radicals) was determined by plotting the percentage inhibition against concentration and calculating using linear regression analysis. The compound is categorized as antioxidant: very strong if IC_50_ < 50 ppm, strong if IC_50_ 50–100 ppm, moderate if IC_50_ 101–150 ppm, and weak if IC_50_ 151–200 ppm.

### 2.4. Antiaging Formulation

Antiaging cream was prepared in 3 formulas containing ethanol extract of Sungkai leaves (*P. canescens*) 0.02% (F1), 0.04% (F2), and 0.06% (F3) as depicted in Table 1. The cream was prepared by mixing the extract and other ingredients such as 12% stearic acid, 5.11% Tween 80, 0.25% Span 80, 4% cetyl alcohol, 18.74% glycerin, 0.09% TEA, 0.20% methylparaben, 0.02% propylparaben, q.s. oleum rosae, and aquadest.

**TABLE 1:** Formulation of Anti Aging Cream.

Ingredient	Function	F1 (%)	F2 (%)	F3 (%)
*P. canescens* extract	Active ingredient	0.02	0.04	0.06
Stearic acid	Emulsifier	12.00	12.00	12.00
Tween 80	Emulsifier	5.11	5.11	5.11
Span 80	Emulsifier	0.25	0.25	0.25
Cetyl alcohol	Thickening agent	4.00	4.00	4.00
Glycerin	Humectant	18.74	18.74	18.74
Triethanolamine (TEA)	pH adjuster	0.09	0.09	0.09
Methylparaben	Preservative	0.20	0.20	0.20
Propylparaben	Preservative	0.02	0.02	0.02
Oleum rosae	Fragrance	q.s.	q.s.	q.s.
Aquadest	Solvent	Ad 100	Ad 100	Ad 100

**FIGURE 4:**
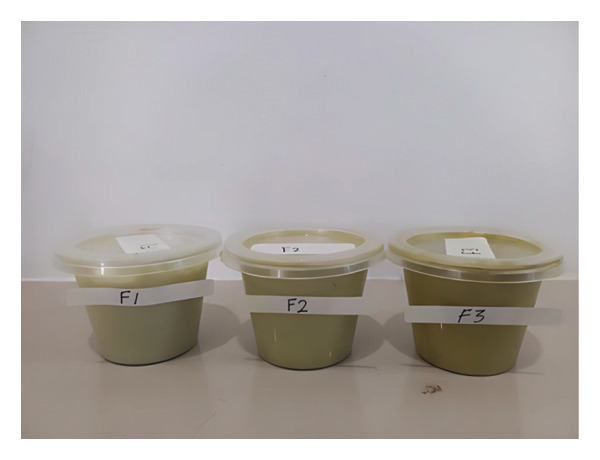
Antiaging cream with Sungkai leaves extract.

The cream was prepared using the standard emulsification method. The oil phase (stearic acid, cetyl alcohol, Span 80) was heated to 70°C in a water bath. The aqueous phase (distilled water, glycerin, Tween 80, TEA) was heated separately to 70°C. The extract was dissolved in a small amount of the aqueous phase. The oil phase was gradually added to the aqueous phase with continuous stirring using a homogenizer (IKA T25 Digital Ultra‐Turrax, IKA‐Werke GmbH, Staufen, Germany) at 2000 rpm for 15 min. Stirring was continued until the mixture cooled to room temperature (approximately 25°C). Preservatives and fragrance were added during the cooling phase at approximately 40°C. The final product was transferred to sterile containers and stored at room temperature, as shown in Figure 4.

### 2.5. Characteristics of Antiaging Cream

The antiaging cream has identified characteristics, including organoleptic, homogeneity, pH, adhesion, and dispersion. The stability of each characteristic was determined by the freeze and thaw method. It storages the cream formula at 4°C for 24 h and then transfers to 40°C for 24 h (1 cycle), subsequently until 6 cycles.

#### 2.5.1. Organoleptic

Organoleptic observation is carried out according to ISO 8586:2012 sensory evaluation standards. A panel of five trained evaluators examined the cream for texture, color, odor, and skin feel. Assessments were conducted before storage (Day 0) and after six freeze–thaw cycles.

#### 2.5.2. Homogeneity

The homogeneity test of the cream was carried out by applying 0.5 g of each cream on a glass slide to observe its homogeneity. The cream was considered homogeneous when no coarse particles were observed on the glass surface.

#### 2.5.3. pH

The pH test was carried out using a calibrated digital pH meter (Mettler Toledo SevenCompact S220, Mettler‐Toledo GmbH, Greifensee, Switzerland). Previously, the electrode was calibrated with pH 4 and pH 7 buffers and then rinsed using distilled water. Next, the electrode was dipped 3 cm deep into each formulation (F1, F2, and F3). Each formula was weighed 1 g and dissolved in 10 mL of distilled water. The pH of the cream must be aligned between 4.5 and 6.5. pH measurements were recorded after stabilization (approximately 2 min). Measurements were performed in triplicate for each formulation weekly during storage for 6 weeks.

#### 2.5.4. Adhesion

The adhesion test aims to determine the ability of the preparation to stick to the skin layer. A quantity of 0.5 g of cream is placed on a glass object slide (25 × 75 mm), covered by another glass object, then given a load of 250 g, allowed to stand for 5 min, and counted the time until the two glass objects are released. The time required for the two glass slides to separate completely was recorded using a digital stopwatch (precision: 0.01 s). Measurements were performed in triplicate before and after storage. Good adhesion was defined as ≥ 4 s.

#### 2.5.5. Dispersion

The dispersion test aims to determine the speed of spreading the cream when applied to the skin surface. A quantity of 0.5 g of cream is placed on a watch glass (diameter: 10 cm) and overwritten with another watch glass and left for 1 min, then given a load (50, 100, 150, 200, and 250 g) each given load allowed to stand for 1 min, then the diameter spreading was measured in two perpendicular directions using a digital caliper (Mitutoyo CD‐15DCX, Mitutoyo Corporation, Kawasaki, Japan; precision: 0.01 mm), and the average was calculated. Measurements were performed in triplicate before and after storage. The acceptable dispersion range was 5–7 cm under a 250 g load.

### 2.6. Statistical Analysis

All measurements were performed in triplicate (*n* = 3) and expressed as mean ± standard deviation (SD). Statistical significance of differences between formulations and before/after storage was assessed using one‐way ANOVA followed by Tukey’s post hoc test using SPSS Statistics Version 26 (IBM Corporation, Armonk, NY, USA). A *p*‐value < 0.05 was considered statistically significant and indicated with an asterisk (∗) in figures and tables.

## 3. Results (and Discussion)

### 3.1. Yield of *P*. *Canescens* Leaves Extract

Based on the results of determining the yield of Sungkai leaf extract, from 250 g of Sungkai leaf simplicia, 45.27 g of crude extract was collected with a yield of 18.10%. This yield is higher than the results of the previous study ([30]; Nurfauziyah et al., 2024b) that using 70% ethanol. The higher the ethanol concentration and the longer the maceration time, the more extracts are obtained. This is because ethanol can react with polar, semipolar, and nonpolar materials, so that the use of ethanol is expected to attract all the content of chemical compounds in Sungkai leaf simplisia.

The relatively high yield obtained in this study can be attributed to several factors, including the polarity of the solvent system used and the extended maceration period. Ethanol at 70% concentration represents an optimal balance between polar and nonpolar solvent properties, enabling efficient extraction of both polar (such as flavonoids, phenolics, and saponins) and semipolar to nonpolar compounds (such as steroids). The addition of 30% water to ethanol enhances the extraction of glycosylated flavonoids and other polar metabolites that may have limited solubility in pure ethanol. Furthermore, the 72‐h maceration period allows sufficient time for cellular disruption and diffusion of phytochemicals from the plant matrix into the solvent, thereby maximizing extraction efficiency.

### 3.2. Chemical Compounds of Extract

Chemical compounds of *P. canescens* leaves (Sungkai leaves) were detected from phytochemical screening analysis. Phytochemical screening results are depicted in Table 2.

**TABLE 2:** Phytochemical screening analysis result.

Test parameters	Reagents	Positive reaction	Result
Flavonoids	Mg powder + concentrated HCl	Orange to red color	+
Phenolic	FeCl3 10%	Blue color	+
Saponins	Shake test	Permanent foam (±15 min)	+
Alkaloids Mayer	Mayer reagent	White precipitate	−
Alkaloids Dragendorff	Dragendorff reagent	Golden yellow color	−
Alkaloids Wagner	Wagner reagent	Reddish brown precipitate	—
Terpenoids	Anhydrous acetic acid + H2 SO4 concentrated + Liebermann–Burchard	Red color	−
Steroids	Anhydrous acetic acid + H2 SO4 concentrated + Liebermann–Burchard	Blue color	+

*Note:* (+) = present; (−) = absent.

Table 2 shows that qualitative flavonoid testing on Sungkai leaf maceration extract showed positive results, indicated by the formation of orange to red color changes after the extract was tested with concentrated HCl and added magnesium ribbon. Flavonoid compounds are polar and extracted in polar solvents which water and ethanol are polar. Therefore, extraction is carried out using solvents that can dissolve components selectively, such as ethanol 70% (30% water). The components contained in the material will be able to dissolve in solvents whose polarity is relatively the same. The use of solvent ionic strength or type of solvent can influence the yield of the resulting compounds [31].

Flavonoids are polyphenolic compounds known for their potent antioxidant properties through multiple mechanisms, including direct free radical scavenging, metal ion chelation, and activation of antioxidant enzymes. In the context of skin aging, flavonoids can protect against photoaging by neutralizing reactive oxygen species (ROS) generated by UV radiation exposure, thereby preventing collagen degradation and maintaining skin elasticity [8].

The results of this study describe that the Sungkai leaf extract showed a positive reaction when reacted with 10% FeCl_3_ solution, and there was a blue color change indicating that the extract contained phenolics. The color changes to blue due to the formation of complex compounds between phenolics and FeCl_3_. Phenolic compounds represent a diverse group of plant secondary metabolites characterized by one or more hydroxyl groups attached to an aromatic ring. These compounds contribute significantly to antioxidant activity through their ability to donate hydrogen atoms to free radicals and stabilize the resulting phenoxyl radicals through resonance delocalization. Moreover, phenolic compounds have been shown to inhibit matrix metalloproteinases (MMPs), enzymes responsible for extracellular matrix degradation during skin aging [17].

The extract also forms permanent foam for ± 15 min, which indicates the content of saponins [26]. The formation of foam after the Sungkai leaf ethanol extract sample was dissolved in warm water and then shaken for 10 s proved the presence of saponin content. Compounds with polar and nonpolar groups are surface active, so when shaken with water, saponins form micelles. In the micelle structure, polar groups face outward because they are hydrophilic while nonpolar groups face inward because they are hydrophobic. This situation looks like foam; from these properties, the presence of saponins in the sample is tested by looking at the ability of the sample to form foam. In cosmetic applications, saponins can function as natural surfactants and may enhance the penetration of other active ingredients through the stratum corneum by interacting with skin lipids.

Testing alkaloid compounds in ethanol extracts of Sungkai leaves by maceration using Mayer’s reagent showed negative results, and no white precipitate was formed. Likewise, the Dragendorff reagent did not change the color of the extract from green to golden yellow, and Wagner’s reagent did not cause a reddish‐brown precipitate. This indicates that the extract does not contain alkaloids [32]. Alkaloid compounds were detected using the Mayer reagent. The presence of alkaloid compounds was indicated by a reaction between nitrogen with potassium ions (K^+^) to form an alkaloid potassium complex that forms a white precipitate [33]. This is because the principle of alkaloid testing using Mayer’s reagent is a precipitation reaction due to ligand replacement. Alkaloid test results using Dragendorff and Wagner reagents also showed negative results.

The absence of alkaloids in our extract contrasts with some previous studies on *P. canescens* [14], which may be attributed to differences in plant maturity, geographical origin, environmental conditions, or extraction methodology.

Negative results were also obtained when the extract was reacted with reagents such as anhydrous acetic acid, concentrated H_2_SO_4_, or Liebermann–Burchard, and the color of the extract did not change to red. However, the color of the extract changed to blue. These results indicated that the extract contains not terpenoids but steroid compounds [25]. This is based on the performance of triterpenoids and steroids to produce color due to the presence of H_2_SO_4_ in anhydrous acetic acid solution. The difference in color between triterpenoids and steroids is due to the difference in groups at the C atom. The principle of reaction in testing terpenoids is condensation or the release of H_2_O and the presence of carbon cation that joins. If there is terpenoid content, the reaction that uses acetic anhydride to acetylate the hydroxyl and acetyl groups will form a double bond [34]. At the next stage, there is a double‐bond displacement due to the release of hydrogen groups and their electrons. The resonance that occurs in this compound works as an electrophile or carbocation. Electrophilic addition is caused by the attack of carbocations which further releases hydrogen, causing the appearance of a brownish ring due to the conjugate extension of the compound due to the hydrogen group leaving along with its electrons [35].

The crude extract tested positive for steroids, as indicated by the blue‐purple color formation with the Liebermann–Burchard reagent. Steroids and steroid‐like compounds in plants, such as phytosterols, have been reported to possess anti‐inflammatory and skin barrier–enhancing properties. They can modulate membrane fluidity and enhance skin barrier function, which is particularly important for aged skin characterized by increased transepidermal water loss [34].

Overall, the phytochemical screening analysis showed that the Sungkai extract in this study contains flavonoids, phenolics, saponins, and steroids. However, alkaloids and terpenoids were not detected in the extract. These findings indicate that the extract has potential antioxidant compounds. The synergistic action of these bioactive compounds may contribute to the observed antioxidant activity and potential skin benefits.

### 3.3. Antioxidant Activity of The Extract

Antiaging cream formula F1 with the least extract in ingredient (0.02% Sungkai extract) was examined for the antioxidant activity. The more extract in the ingredient, the more antioxidant activity. Thus, one sample of the formula is enough to be examined. The antioxidant activity of formula cream (F1) and comparison to Vitamin C at control absorbance 0.546 ± 0.008 A is depicted in Table 3.

**TABLE 3:** Antioxidant activity of Sungkai leaves extract and Vitamin C.

Sample	Concentration (ppm)	The absorbance of sample + DPPH (A)	Inhibition (%)	IC50 (ppm)
Formula F1	10	0.341	37.54	21.98 (very strong)
	20	0.288	47.25	
	30	0.224	58.97	
	40	0.172	68.49	
	50	0.102	81.31	

Vitamin C	2	0.323	40.84	4.12 (very strong)
	4	0.274	49.81	
	6	0.231	57.69	
	8	0.181	66.84	
	10	0.142	73.99	

*Note:* Control absorbance (DPPH + ethanol) = 0.546 ± 0.008 A; values are expressed as mean ± SD (*n* = 3).

Rationale for testing only F1 for antioxidant activity: Only formulation F1 (containing 0.02% extract) was tested for antioxidant activity because the antioxidant capacity is directly proportional to extract concentration. Since formulations F2 (0.04%) and F3 (0.06%) contain 2 × and 3 × the extract concentration of F1, respectively, their antioxidant activities would predictably be proportionally stronger. Testing F1 provides the baseline antioxidant activity, which is scientifically valid as the relationship between extract concentration and antioxidant activity has been well established in the concentration‐response curve. This approach is cost‐effective while all three formulations were comprehensively evaluated for their physicochemical properties.

The results showed that with the increase in extract concentration, the absorbance of the sample will decrease and the inhibition level will increase. The absorbance of the sample decreased because the electrons on DPPH bind to the electrons on the H atom owned by the sample resulting in the color of the solution changing from concentrated purple to clear yellow. The inhibition level value increases as the sample concentration increases due to more antioxidant compounds in the sample that can counteract free radicals [24].

Antioxidant activity test was conducted using DPPH method, where DPPH (a purple colored compound) was used as a free radical indicator. The results showed that ethanol extract of Sungkai leaves can capture DPPH free radicals. The higher the concentration of the extract used, the higher the level of inhibition, which is reflected in a decrease in the absorbance of the DPPH solution and a change in the color of the solution from purple to yellow (Dai et al., 2023).

Table 3 shows that the cream formulation obtained IC_50_ of 21.98 ± 1.23 ppm. Thus, cream formulation from Sungkai leaves extract has very strong antioxidant activity. IC_50_ is a value that indicates the concentration inhibits free radicals by 50% [6]. The smaller the IC_50_ value, the higher the free radical scavenging activity. However, antioxidants activity of Vitamin C (4.12 ± 0.34 ppm) is approximately 5.3 times higher than cream formula F1 (21.98 ± 1.23 ppm). This is consistent with vitamin C being one of the most powerful natural antioxidants and a standard reference compound in antioxidant assays.

The DPPH radical scavenging assay is based on the reduction of the stable DPPH radical (purple color, λmax = 517 nm) to the nonradical form DPPH‐H (yellow color) by hydrogen or electron donation from antioxidant compounds. The results clearly demonstrate a concentration‐dependent increase in antioxidant activity, with higher extract concentrations producing greater DPPH radical scavenging (lower absorbance and higher inhibition percentage).

Referring to phytochemical screening results, the presence of flavonoids, phenolics, saponins, and steroids in the extracts supported the findings of antioxidant activity. These compounds are known to have the potential to act as antioxidants by capturing free radicals [19]. The strong antioxidant activity observed can be attributed to the presence of these bioactive compounds. Flavonoids contribute to antioxidant activity through several mechanisms: (1) direct scavenging of free radicals via donation of hydrogen atoms from hydroxyl groups, (2) chelation of pro‐oxidant metal ions such as Fe^2+^ and Cu^2+^, and (3) inhibition of oxidative enzymes. Phenolic compounds similarly donate hydrogen atoms to neutralize free radicals, with the resulting phenoxyl radicals being stabilized by resonance delocalization across the aromatic ring system. Therefore, the relationship between the phytochemical composition of the extract and antioxidant activity is a determining factor in the effectiveness of Sungkai leaf extract as an antioxidant agent.

The IC_50_ value obtained in this study (21.98 ppm) is lower (indicating stronger activity) than previously reported values for *P. canescens* leaf extracts by Latief et al. [36], who reported IC_50_ values of 50.838 ppm for young leaves and 52.835 ppm for old leaves. This difference may be explained by several factors: (1) variations in extraction efficiency due to different extraction parameters (solvent concentration, extraction time, and temperature), (2) seasonal or geographical variations in phytochemical content, (3) differences in leaf maturity at harvest, and (4) variations in assay conditions. Our optimized extraction protocol using 70% ethanol for 72 h appears to have maximized the recovery of antioxidant compounds.

### 3.4. Organoleptic of the Cream Formula

Organoleptic including texture, color, smell, and skin feel is presented in Table 4.

**TABLE 4:** Organoleptic of Sungkai leaves antiaging cream formula.

Formula	Organoleptic test	Before storage	After storage
F1	Texture	Emulsion	Emulsion
	Color	Pale‐green	Pale‐green
	Smell	Authentic	Authentic
	Skin feel	Cool	Cool

F2	Texture	Cream	Cream
	Color	Light Green	Light Green
	Smell	authentic	authentic
	Skin feel	Cool	Cool

F3	Texture	Cream	Cream
	Color	Green	Green
	Smell	Authentic	Authentic
	Skin feel	Cool	Cool

*Note:* Organoleptic evaluation was performed by a trained sensory panel (*n* = 5) according to ISO 8586:2012; texture descriptions: smooth (easy to spread), nonsticky (does not leave residue), slightly oily (moderate emollient effect), oily (rich emollient effect); skin feel descriptions: rapidly absorbed (quick penetration), moderately absorbed (balanced penetration), slowly absorbed (longer surface contact), silky (smooth finish), and moisturizing (hydrating effect).

Organoleptic showed that antiaging formula from Sungkai leaves extract before storage was the same as after storage. Therefore, the organoleptic of the antiaging formula was stable. All three formulations maintained consistent organoleptic properties before and after six freeze–thaw cycles, indicating good stability under temperature stress conditions.

The texture progressively changed from a light emulsion (F1) to a thick cream (F3) with increasing extract concentration, which is attributed to the viscosity‐enhancing effect of the phytochemical constituents in the extract. The color intensified from pale green to green with higher extract concentrations, reflecting the natural chlorophyll and other pigments present in the *P. canescens* leaf extract. This color variation is acceptable and may even be perceived as desirable by consumers seeking natural, plant‐based cosmetic products.

The characteristic herbal scent remained consistent across all formulations and did not change during storage, indicating absence of oxidative degradation or microbial contamination. The cooling sensation upon application, likely attributable to the evaporation of water and volatile components, was appreciated by the sensory panel and is a desirable attribute for skincare products. The skin feel varied among formulations: F1 provided a light, rapidly absorbed finish suitable for oily or combination skin types; F2 offered a balanced texture appropriate for normal skin; and F3 delivered a richer, more moisturizing experience ideal for dry or mature skin.

### 3.5. Homogeneity of the Cream Formula

The homogeneity of cream formulas F1, F2, and F3 is presented in Table 5.

**TABLE 5:** Homogeneity of Sungkai leaves antiaging cream formula.

Cream formula	Before storage	After storage
F1	Homogenous	Homogenous
F2	Homogenous	Homogenous
F3	Homogenous	Homogenous

*Note:* Homogeneity was assessed visually on glass slides under natural light; absence of coarse particles, phase separation, or aggregates indicates good homogeneity.

After the cream formulations containing the Sungkai leaf extract were spread on a glass slide before storage, no coarse particles were observed on the glass surface in F1, F2, or F3. It is shown that cream formulas F1, F2, and F3 were homogenous before storage. Coarse grains were not detected on the glass surface when cream formulas F1, F2, and F3 (after storage) were spread on the glass. It is shown that storage did not change the cream formulas F1, F2, and F3.

All three formulations exhibited excellent homogeneity both before and after storage, with no visible coarse particles, phase separation, or aggregation observed. This indicates that the emulsification process was successful and that the emulsifying agents (stearic acid, Tween 80, Span 80, and cetyl alcohol) were effective in creating stable oil‐in‐water emulsions. Homogeneity is critical for ensuring consistent dosing of active ingredients, uniform spreading upon application, and esthetic appeal.

### 3.6. pH of the Cream Formula

pH of 1 g cream formula dissolved into 10 mL of aquadest is depicted in Table 6.

**TABLE 6:** pH of Sungkai leaves antiaging cream formula.

Cream formula	Week						Average
	1	2	3	4	5	6	
F1	5.62	5.51	5.43	5.54	5.61	5.51	5.54
F2	5.43	5.17	5.24	5.11	4.93	5.10	5.16
F3	4.99	5.03	5.00	4.95	5.08	5.00	5.01

*Note:* Values are expressed as mean ± SD (*n* = 3); *p* < 0.05 compared to F1 (one‐way ANOVA with Tukey’s post hoc test); acceptable pH range for topical skincare is 4.5–6.5.

Cream formula F1 from Week 1 to Week 6 was within an average pH of 5.54 ± 0.06; F2 average pH of 5.16 ± 0.12, and F3 average pH of 5.01 ± 0.05. Those pH were in the range of 4.5–6.5. This means that the cream formula has a stable pH for storage for 6 weeks at room temperature. pH less than 4.5 or strongly acidic gives irritation to the skin; meanwhile, pH more than 6.5 or strongly alkaline gives scaly skin [37].

All three formulations maintained pH values within the acceptable range for topical skincare products throughout the 6‐week storage period. The skin’s natural pH (acid mantle) ranges from 4.5 to 6.0, with an average of approximately 5.5. Formulations within this range are considered skin‐compatible and minimize the risk of irritation or disruption of the skin’s natural barrier function.

Formulation F1 exhibited the highest average pH (5.54 ± 0.06), which is very close to the skin’s natural pH and showed minimal variation during storage, indicating excellent pH stability. Formulations F2 (5.16 ± 0.12) and F3 (5.01 ± 0.05) showed progressively lower pH values, which is attributed to the higher concentration of *P. canescens* extract containing acidic phenolic and flavonoid compounds. The slight acidity may actually be beneficial, as mildly acidic formulations can enhance skin barrier function. The statistical analysis revealed significant differences (*p* < 0.05) between F2, F3, and F1, indicating that extract concentration influences the final pH of the formulation.

### 3.7. Adhesion of the Cream Formula

**FIGURE 5:**
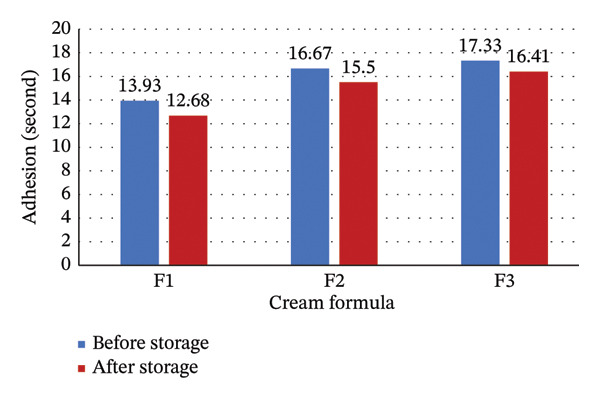
Adhesion of cream formula. Data are expressed as mean ± SD (*n* = 3). ^∗^ *p* < 0.05 compared to before storage (paired *t*‐test). All formulations met the minimum adhesion requirement (> 4 s).

The adhesion of cream formulation from Sungkai leaves extract is presented in Figure 5.

The adhesion of formula F1 (13.93 ± 0.88 and 12.68 ± 0.76 s), F2 (16.67 ± 1.02 and 15.50 ± 0.94 s), and F3 (17.33 ± 1.15 and 16.41 ± 1.08 s) met the standard (more than 4 s) [1, 7]. This means that the cream formula has good adhesion. The adhesion test in Figure 5 shows that adhesion of F3 is significantly longer than F2 and F1 (*p* < 0.05). Thus, the higher the concentration of Sungkai leaves extract in the cream formula, the significantly longer the adhesion. The longer adhesion allows the extract of Sungkai leaves to adsorb better into the skin because the active substances released on the base of the cream will be absorbed more [38], which can produce the desired effect.

However, the adhesion of the cream formula after storage was significantly decreased for all formulations (F1, F2, and F3) (*p* < 0.05). The decreases ranged from 9.0% for F1 to 5.3% for F3. This means that the cream formula was not stable enough in adhesion. This could be attributed to (1) partial coalescence of the emulsion droplets leading to microstructural reorganization, (2) weakening of the interfacial film around oil droplets, and (3) changes in the hydrogen bonding network between formulation components. Thus, it needs improvement for better stability in adhesion. Future formulation development could consider incorporation of polymeric thickening agents with better freeze–thaw stability or optimization of the emulsifier system.

### 3.8. Dispersion of the Cream Formulation

**FIGURE 6:**
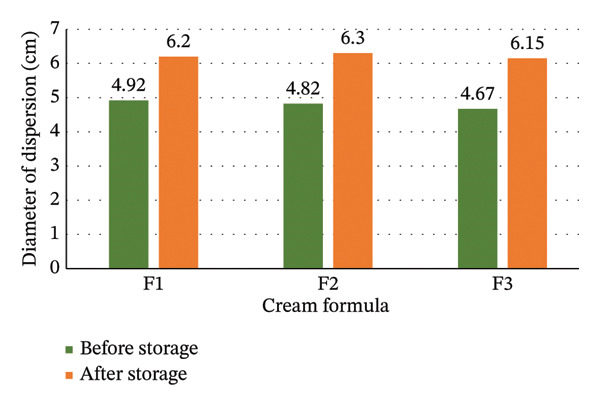
Dispersion of cream formulations before and after storage under 250 g load. Data are expressed as mean ± SD (*n* = 3). ^∗^ *p* < 0.05 compared to before storage (paired *t*‐test). Acceptable dispersion range is 5–7 cm.

Dispersion of each formula (F1, F2, and F3) is presented in Figure 6.

The dispersion of formula F1 (4.92 ± 0.15 and 6.20 ± 0.22 cm), F2 (4.82 ± 0.18 and 6.30 ± 0.25 cm), and F3 (4.67 ± 0.12 and 6.15 ± 0.20 cm) met the standard (5–7 cm) [1, 7]. This means that the cream formula has good dispersion. The dispersion test in Figure 6 shows that the dispersion of F3 (4.67 ± 0.12 cm) is significantly smaller than F2 (4.82 ± 0.18 cm) and F1 (4.92 ± 0.15 cm) (*p* < 0.05). Thus, the higher the concentration of Sungkai leaves extract in the cream formula, the significantly smaller the dispersion. The higher concentration of Sungkai leaves extract produces a thicker cream, thus, the smaller dispersion [39]. This is consistent with the adhesion results, as more viscous formulations tend to exhibit both longer adhesion times and reduced spreadability.

However, the dispersion of the cream formula after storage significantly increased for all formulations (F1, F2, and F3) (*p* < 0.05). The increases ranged from 26.0% for F1 to 31.7% for F3. While the initial dispersion values for F1 and F2 were slightly below the acceptable range (5–7 cm), all formulations fell within the acceptable range after storage. This means that the cream formula was not stable enough in dispersion. This phenomenon can be explained by (1) partial breakdown of the emulsion structure leading to reduced internal phase interactions, (2) disruption of the three‐dimensional network formed by thickening agents, and (3) reduction in hydrogen bonding between ingredients. Thus, it needs improvement for better dispersion stability. Future formulation optimization could include incorporation of rheology modifiers with better freeze–thaw stability or use of mixed emulsifier systems.

It is noteworthy that while both adhesion and dispersion showed statistically significant changes after storage, the formulations still met the minimum quality requirements. This demonstrates that *P. canescens* extract can be successfully formulated into functional antiaging creams, although further optimization is needed to achieve optimal long‐term stability.

## 4. Conclusion

The ethanol extract of *P. canescens* Jack. (Sungkai leaves) from one of the regencies in West Sumatera, Indonesia, has good characteristics as antiaging ingredient in skincare. Based on the phytochemical screening analysis, it positively contains secondary metabolite compounds of flavonoids, phenolics, saponins, and steroids. It also has a very strong antioxidants activity, IC_50_ of 21.98 ± 1.23 ppm. This study has formulated the antiaging cream with various extract concentrations (0.02%, 0.04%, and 0.06%).

The cream formulas have good characteristics, including organoleptic, homogeneity, pH, adhesion, and dispersions. All formulations exhibited acceptable pH values (F1: 5.54 ± 0.06, F2: 5.16 ± 0.12, and F3: 5.01 ± 0.05) within the optimal range (4.5–6.5), excellent homogeneity with no phase separation, adequate adhesion times (> 4 s), and appropriate dispersion values (5–7 cm) for easy application.

In conclusion, antiaging cream formula of the *P. canescens* Jack. (Sungkai leaves) extract has been produced with good results. However, the cream formulas have limitations such as insufficient stability in adhesion and dispersion. Both adhesion and dispersion properties showed statistically significant changes after six freeze–thaw cycles: adhesion decreased by 5.3%–9.0% (*p* < 0.05), while dispersion increased by 26.0%–31.7% (*p* < 0.05). These changes indicate areas requiring formulation optimization using alternative stabilizers, thickening agents, or mixed emulsifier systems to enhance long‐term stability.

This study contributes significance to understanding the characteristics and potential of Sungkai leaves as anti‐ageing ingredients and could be applied as a basis for further in‐depth study. The findings provide a foundation for developing sustainable, plant‐based skincare products that combine traditional botanical knowledge with modern cosmetic science, potentially benefiting both the cosmetics industry and local communities through valorization of indigenous plant resources. Future studies should focus on clinical efficacy evaluation, long‐term stability testing under ICH guidelines, safety assessment, and mechanistic studies on cellular effects of the extract on skin aging pathways.

## Author Contributions

Rahmiati: conceptualization, funding acquisition, writing–original draft, writing–review and editing, and reviewed the manuscript. Ringga Novelmi: investigation, resources, software, and reviewed the manuscript. Vivi Efrianova and Febri Silvia: methodology, writing–review and editing, and reviewed the manuscript. Tyas Asih Surya Mentari: visualization, data curation, and reviewed the manuscript. Siska Miga Dewi: project administration, resources, and reviewed the manuscript. Febri Silvia: methodology, software, writing–review and editing, and reviewed the manuscript. Hayatunnufus: data curation, writing–original draft, and reviewed the manuscript. Rahmi Oktarina: validation and data curation. Dony Novaliendry: data collection, preparation of the original draft, and finalization of the manuscript through review and editing. Merita Yanita: formal analysis, data curation, and reviewed the manuscript. Murni Astuti: supervision, data curation, and reviewed the manuscript.

## Funding

No funding was received for this manuscript.

## Conflicts of Interest

The authors declare no conflicts of interest.

## Data Availability

The datasets used and/or analyzed during the current study are available from the corresponding author upon reasonable request.
